# Design and Optimization of LTE 1800 MIMO Antenna

**DOI:** 10.1155/2014/725806

**Published:** 2014-05-20

**Authors:** Huey Shin Wong, Mohammad Tariqul Islam, Salehin Kibria

**Affiliations:** ^1^Center for Space Science, Universiti Kebangsaan Malaysia (UKM), 43600 Bangi, Malaysia; ^2^Department of Electrical, Electronic and Systems Engineering, Faculty of Engineering and Built Environment, Universiti Kebangsaan Malaysia (UKM), 43600 Bangi, Malaysia

## Abstract

A multiple input and multiple output (MIMO) antenna that comprises a printed microstrip antenna and a printed double-L sleeve monopole antenna for LTE 1800 wireless application is presented. The printed double-L sleeve monopole antenna is fed by a 50 ohm coplanar waveguide (CPW). A novel T-shaped microstrip feedline printed on the other side of the PCB is used to excite the waveguide's outer shell. Isolation characteristics better than −15 dB can be obtained for the proposed MIMO antenna. The proposed antenna can operate in LTE 1800 (1710 MHz–1880 MHz). This antenna exhibits omnidirectional characteristics. The efficiency of the antenna is greater than 70% and has high gain of 2.18 dBi.

## 1. Introduction

In recent years, advances in wireless technology have led to the insatiable demand for wireless broadband. The LTE standard can solve this problem by supporting higher data rates, higher capacity, and lower latency [1–3]. LTE 1800 has gained a lot of interests among wireless broadband operators. This is primarily due to the 1800 MHz band that is already being used for GSM 1800. The spectrum refarming from GSM 1800 to LTE 1800 is very cost effective. A lot of researches have been done to develop LTE antennas [4, 5], but there is lack of research for LTE 1800 MIMO antenna. As the deployments of LTE 1800 continue to accelerate, the development and optimization of LTE 1800 antenna are beneficial to meet the modern demands of wireless terminals.

Printed sleeve monopole antennas are low profile with its planar structure. The sleeves that are added to the ground plane of the monopole antenna act as a parasitic element to generate additional resonant mode [6]. This additional resonant mode combines with the fundament resonant mode to generate wide bandwidth. Various types of sleeves have been proposed such as L-shaped sleeves [7] and tilted sleeves [8].

Several challenges are faced in order to integrate multiple antennas into a laptop. One of the main challenges in MIMO antenna design is to obtain good isolation characteristics between two antennas [9]. In order to reduce mutual coupling between multiple antennas, a lot of research has been done in order to overcome this challenge. In [10], a dual feed single element antenna for 4G MIMO devices is proposed. Isolated mode antenna technology is used to reduce the mutual coupling between the two ports. It occupies an area of 88.4 × 64.2 mm^2^. In this paper, the proposed antenna is a combination of printed microstrip and a printed double-L sleeve monopole antenna. This proposed antenna can cover LTE 1800 frequency band for laptop or tablets application. It has a smaller size as compared to [10]. The structure of the proposed antenna is described in detail in the following section. The effects of the varying parameters of the proposed MIMO antenna on the antenna performance are also presented in this paper.

## 2. Antenna Design

**Figure 1:**
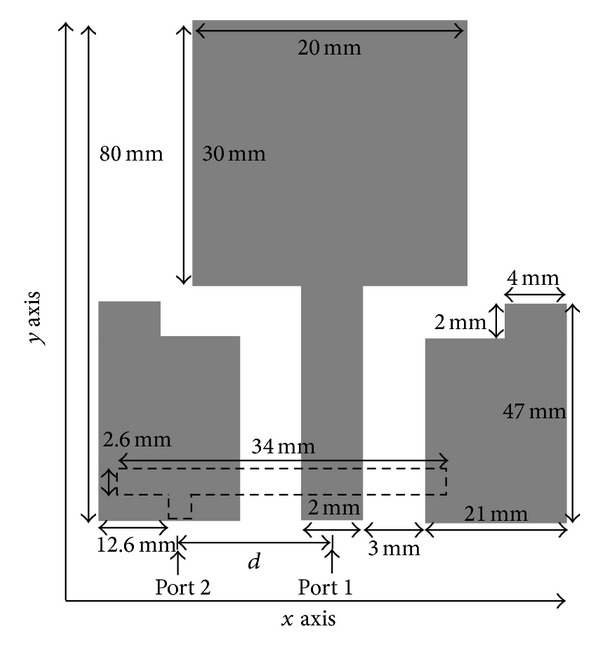
Structure and dimension of proposed MIMO antenna.

**Figure 2:**
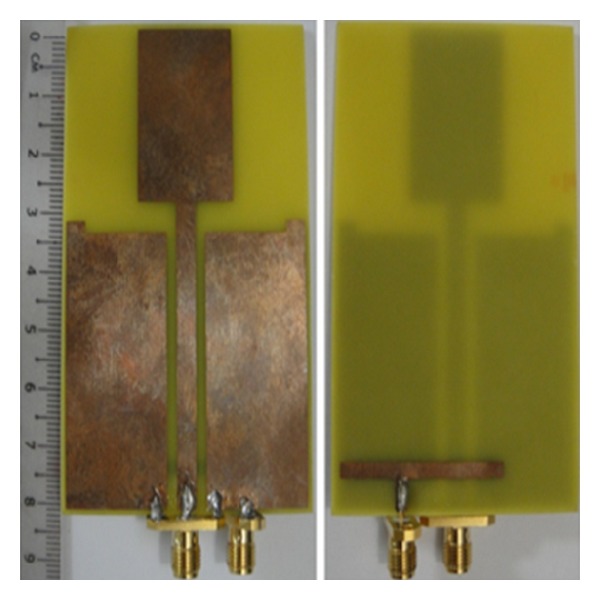
Front and back view of prototype MIMO antenna.

The proposed antenna design as shown in Figure 1 occupies the size of 80 × 50 mm^2^. The material chosen for the antenna is a FR4 substrate with dielectric permittivity of 4.6 and thickness of 1.6 mm.  Figure 2 shows the front and back view of the prototyped antenna. A printed double-L sleeve monopole antenna is printed on the front side of the printed circuit board (PCB). Two symmetrical ground planes are located at the bottom of the PCB surrounding the printed monopole. The edges of the ground plane are extended to form an L-shaped ground plane. A CPW is used to feed the printed double-L sleeve monopole antenna at Port 1. A SubMiniature version A (SMA) connector is soldered to the 50 ohm CPW. The two-symmetrical ground planes at the bottom of PCB are connected by the SMA connector. A T-shaped microstrip feedline is printed on the backside of the PCB. The T-shaped microstrip feedline is used to excite the waveguide's outer shell on the other side of the PCB. The length of the feedline, 34 mm, is 81.6% of quarter wavelength at 1800 MHz. The T-shaped microstrip feedline is fed at 11.4 mm from the left end of the feedline at Port 2. It is a microstrip monopole with offset fed antenna. The distance between Port 1 and Port 2 is 11.3 mm.

As shown in Figure 1, the printed double-L sleeve monopole antenna consists of a printed monopole in the middle and two-symmetrical L-shaped sleeves at the sides. The transmission line model method is used to determine the dimensions of the printed monopole to achieve the desired frequency. The double L-shaped sleeve acts as a parasitic element to improve the bandwidth of the printed monopole antenna. A T-shaped microstrip feedline is printed on the other side of the PCB. The T-shaped feedline is completely covered by the ground plane on the other side of the PCB. This structure allows efficient radiation properties.

**Figure 3:**
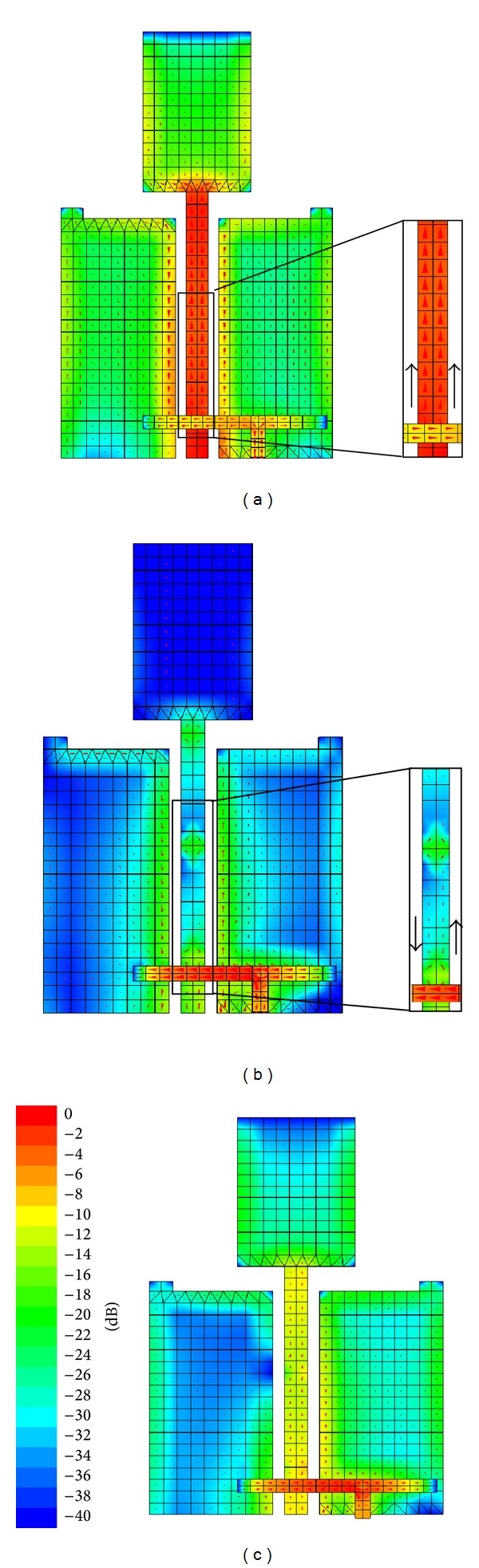
(a) Surface current distribution at 1800 MHz of the printed double-L sleeve monopole antenna only, (b) surface current distribution at 1800 MHz of the T-shaped microstrip feedline antenna only, and (c) surface current distribution at 1800 MHz of the proposed MIMO antenna.

The combination of printed double-L sleeve monopole antenna and a T-shaped microstrip feedline antenna is chosen mainly because of current distribution characteristics. The structure of the printed double-L sleeve monopole antenna is designed to be symmetrical. A CPW is located at the symmetrical line of the printed double-L sleeve monopole antenna. The current distribution for the printed double-L sleeve monopole antenna is in phase and of equal magnitude. The current distribution is out of phase for the T-shaped microstrip feedline antenna.  Figure 3 shows the current distribution at 1800 MHz of the printed double-L sleeve monopole antenna only, T-shaped microstrip feedline antenna only, and the proposed MIMO antenna. As shown in Figure 3(a), when only the printed double-L sleeve monopole antenna is excited, the currents at the CPW are flowing in an upward direction. On the other hand, when only the T-shaped microstrip feedline antenna is excited, the currents at the CPW are flowing in circular loop as shown in Figure 3(b). This allows both modes to exist simultaneously and independently of each other, resulting in low coupling between the two ports. In Figure 3(b), high concentration of currents can be observed at the T-shaped microstrip feedline. This leads to coupled vertical currents at the printed double-L sleeve monopole antenna. Vertical currents generated at the L-shaped ground plane on the left side of the printed double-L sleeve monopole antenna are in the upward direction. On the other hand, vertical currents generated at the L-shaped ground plane on the right side of the printed double-L sleeve monopole antenna are in the downward direction. The current flows at the left and right side of printed double-L sleeve monopole are in opposite direction. Hence, it does not lead to any net current flow into Port 1. Overall, good isolation characteristics between Port 1 and Port 2 can be achieved.

**Figure 4:**
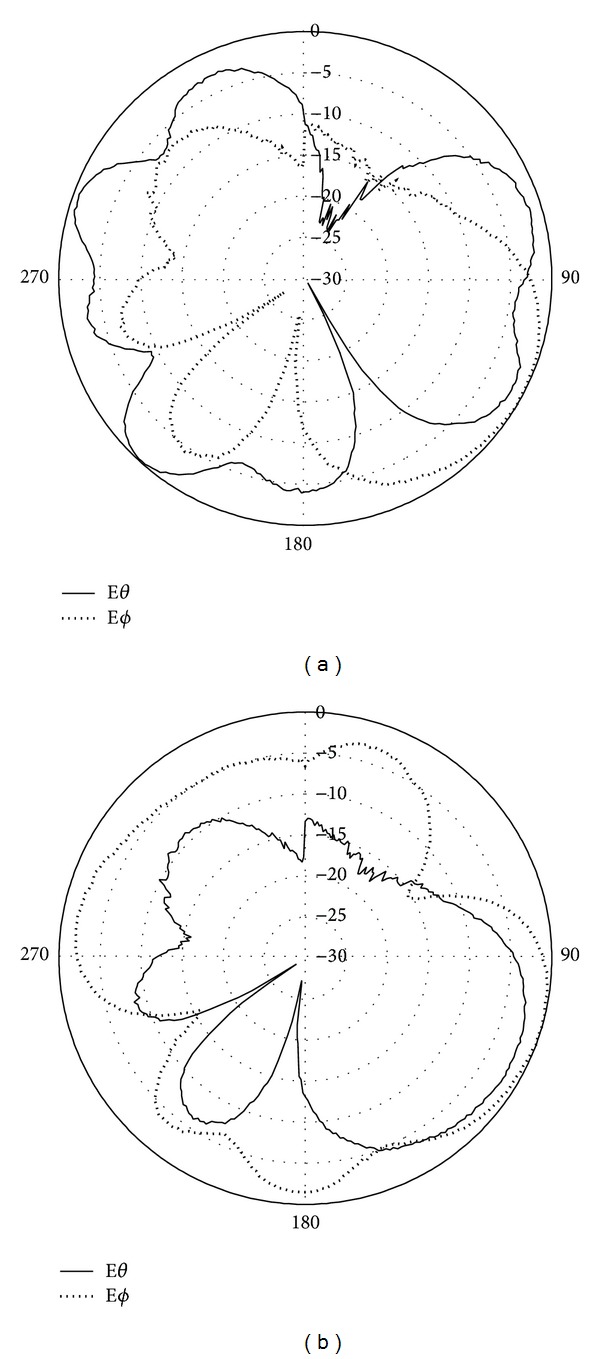
Simulated radiation patterns at 1800 MHz for printed double-L sleeve monopole antenna only (a) E-plane and (b) H-plane.

**Figure 5:**
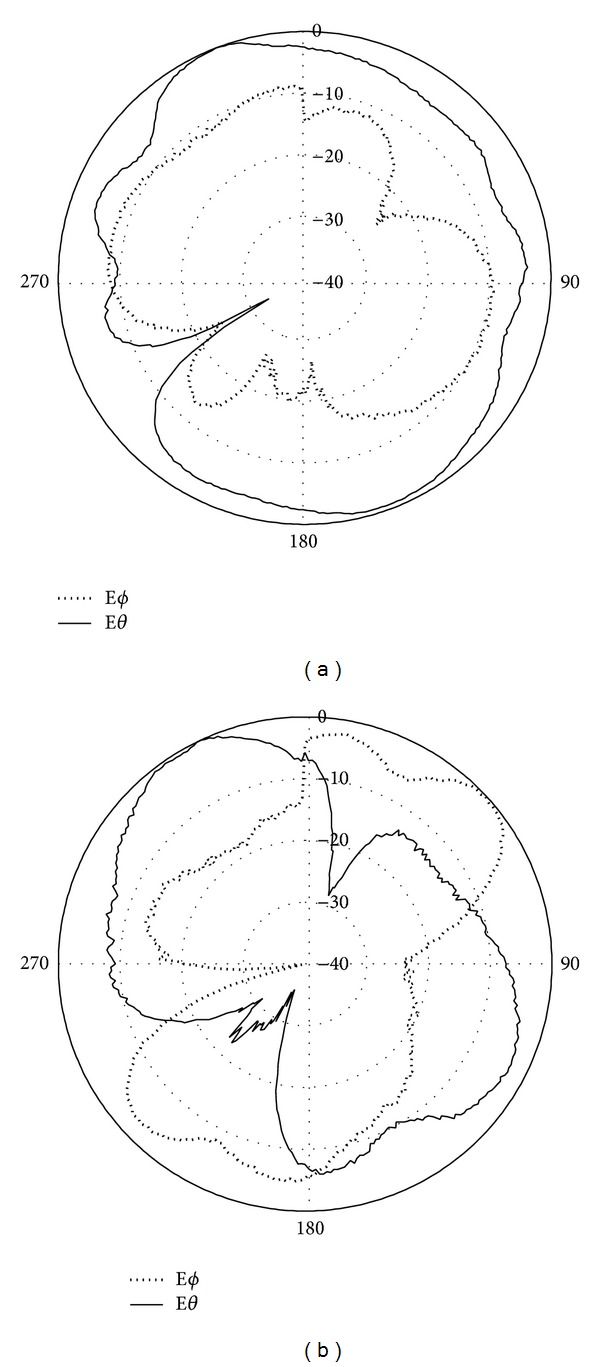
Simulated radiation patterns at 1800 MHz for T-shaped microstrip feedline antenna only (a) E-plane and (b) H-plane.

Figures 4(a) and 4(b) illustrate the radiation pattern at 1800 MHz for E-plane and H-plane of the printed double-L sleeve monopole antenna, respectively. In Figure 4(a), E-phi and E-theta for the E-plane are given. For H-plane, the E-theta and E-phi are illustrated in Figure 4(b). The radiation patterns for T-shape microstrip feedline antenna at 1800 MHz are depicted in Figures 5(a) and 5(b). In Figure 5(a), the E-theta and E-phi for E-plane are shown. The E-theta and E-phi for the H-plane are given in Figure 5(b).

## 3. Results and Analysis

**Figure 6:**
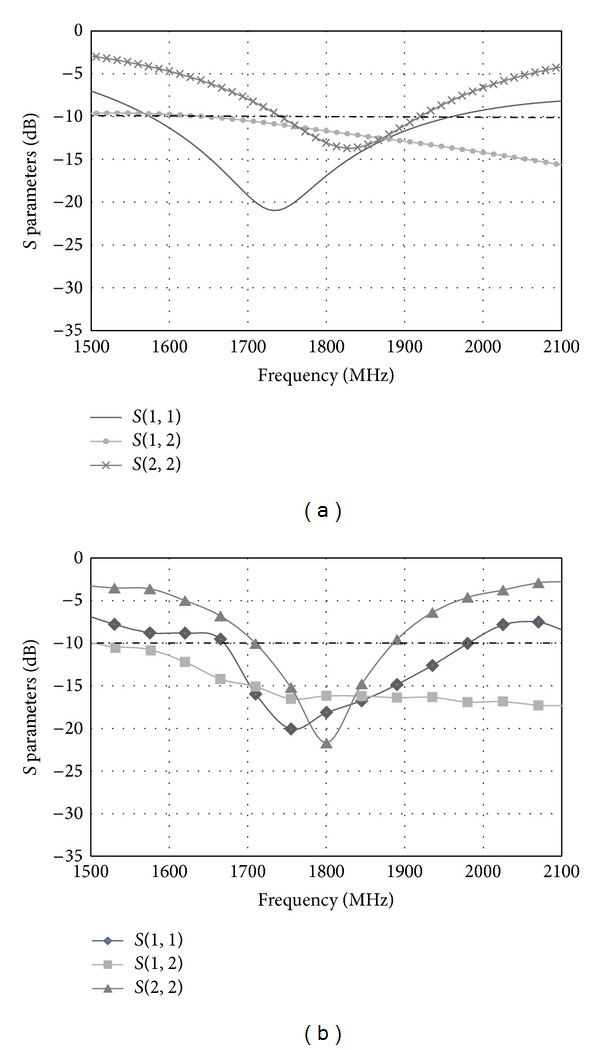
(a) Simulated *S* parameters of the proposed MIMO antenna. (b) Measured *S* parameters of the proposed MIMO antenna.

**Figure 7:**
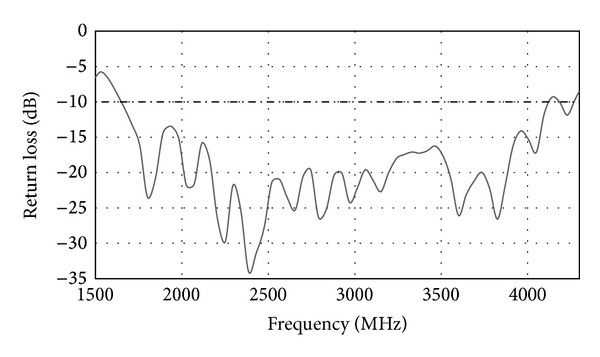
Measured return loss with printed double-L sleeve monopole antenna only.

**Figure 8:**
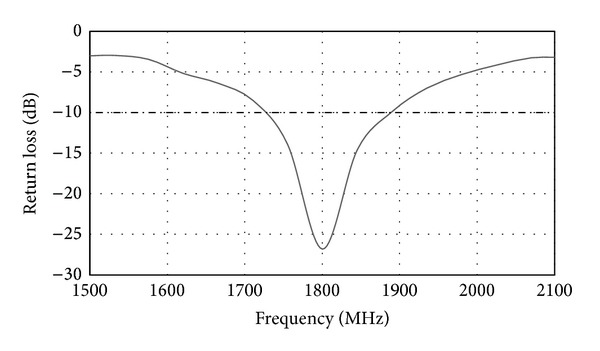
Measured return loss with T-shaped microstrip feedline antenna only.

The proposed antenna is simulated using IE3D.  Figure 6 shows the simulated and measured results (*S*_11_, *S*_21_, and *S*_22_) of the MIMO antenna. The differences in *S* parameters between the measured results and the simulated results are due to the imperfections during the fabrication process. From the measured results, the frequency range is from 1710 MHz to 1880 MHz at the return loss 10 dB. A bandwidth of 170 MHz is obtained. At 1800 MHz, the isolation between Port 1 and Port 2 is about −16.17 dB. In Figure 7, the measured *S* parameter (*S*_11_) for only the printed double-L sleeve monopole antenna is shown. The printed double-L sleeve monopole antenna has a wide operating frequency range from 1680 MHz to 4230 MHz. The measured *S* parameter (*S*_11_) for the T-shaped microstrip feedline antenna only is shown in Figure 8. Taking the return loss of 10 dB, the T-shaped microstrip feedline antenna can operate from 1710 MHz to 1880 MHz.

**Figure 9:**
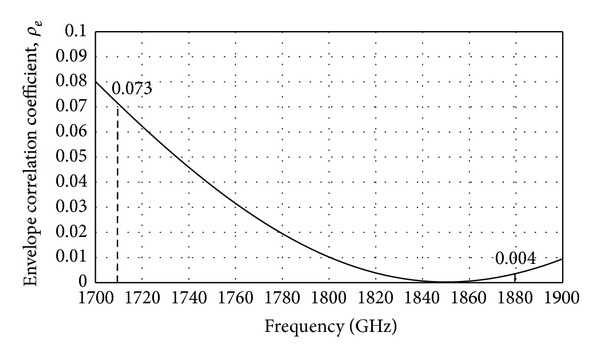
Envelope correlation coefficient, *ρ*_*e*_ of the proposed MIMO antenna.

**Figure 10:**
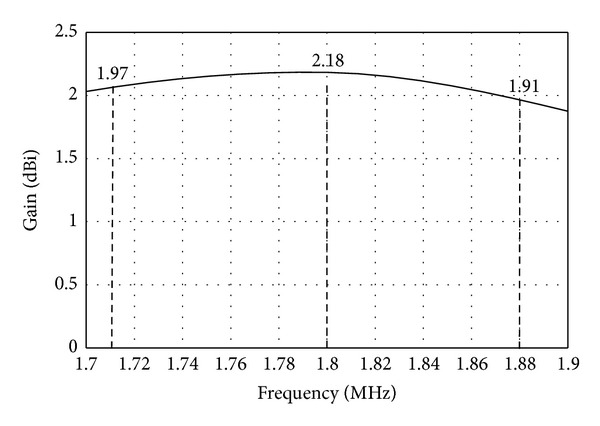
Simulated gain of the proposed MIMO antenna.

**Figure 11:**
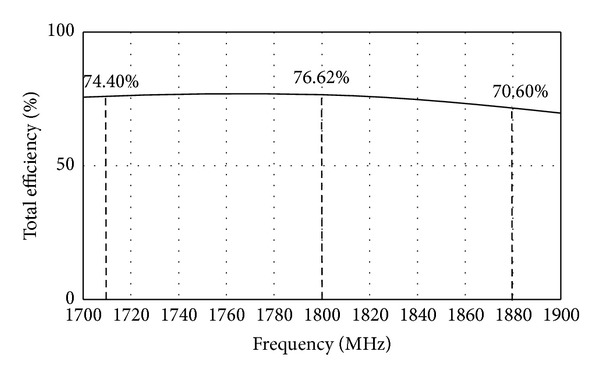
Simulated total efficiency of the proposed MIMO antenna.

Envelope correlation coefficient (*ρ*_*e*_) is used to show the diversity capabilities of a MIMO system [11]. The formula given in (1) is used to calculate the *ρ*_*e*_ of a dual antenna MIMO system [12]. The calculated envelope correlation coefficient of the proposed MIMO antenna is given in Figure 9. It can be observed that the proposed antenna has an envelope correlation coefficient of less than 0.07 over the LTE 1800 band.This is acceptable for MIMO applications [13, 14]:

(1) $$\begin{array}{c} \rho_{e}=\frac{{\left|S_{11}^{*}S_{12}+S_{21}^{*}S_{22}\right|}^{2}}{\left[1-\left({\left|S_{11}\right|}^{2}+{\left|S_{21}\right|}^{2}\right)\right]\left[1-\left({\left|S_{22}\right|}^{2}+{\left|S_{12}\right|}^{2}\right)\right]} \end{array}$$

the proposed antenna has high gain and high efficiency. At 1800 MHz, the antenna gain is the highest with 2.18 dBi as shown in Figure 10.  Figure 11 shows simulated total efficiency of the proposed MIMO antenna. The total efficiency at the LTE1800 band (1710 MHz–1880 MHz) varies from 74.40% to 70.60%. At the resonance frequency, 1800 MHz, the total efficiency is 76.62%.

**Figure 12:**
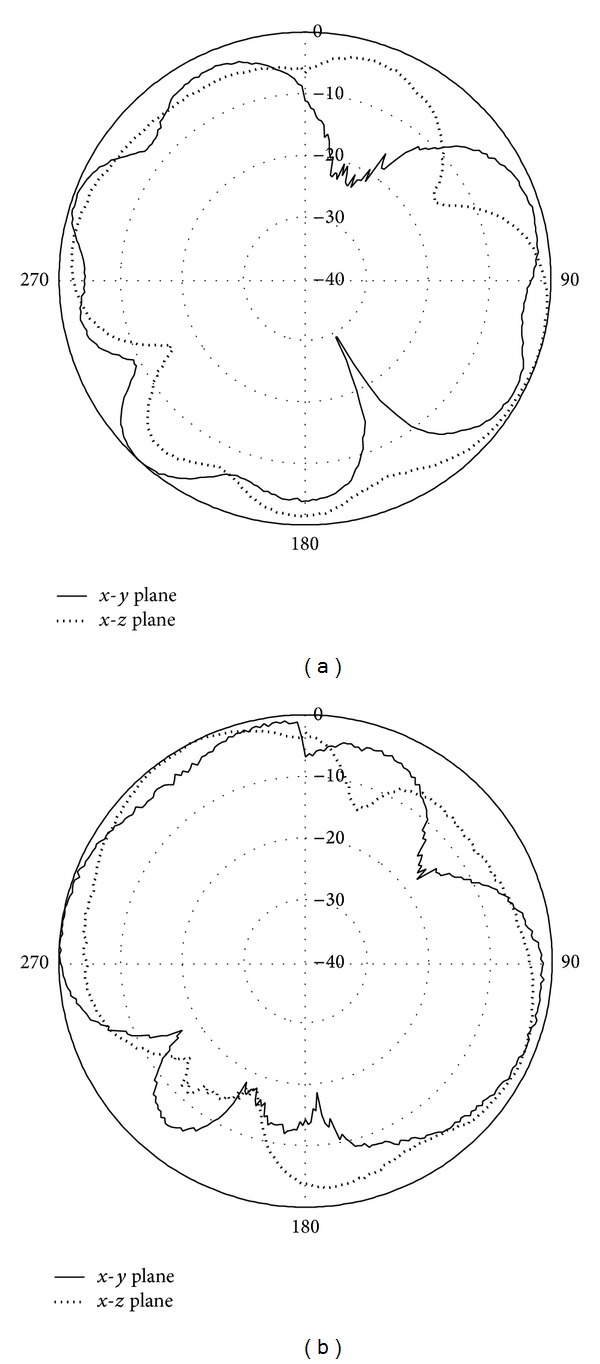
(a) Measured radiation patterns at 1800 MHz for Port 1 antenna. (b) Measured radiation patterns at 1800 MHz for Port 2 antenna.

The measured radiation patterns at the frequency 1800 MHz are shown in Figure 12. In Figure 12(a), the radiation pattern for the printed double-L sleeve monopole antenna is shown. It can be observed that the radiation pattern of the Port 1 antenna is omnidirectional.  Figure 12(b) shows the measured radiation pattern for Port 2 antenna. The radiation pattern for the T-shaped microstrip feedline antenna is approximately an omnidirectional pattern.

**Figure 13:**
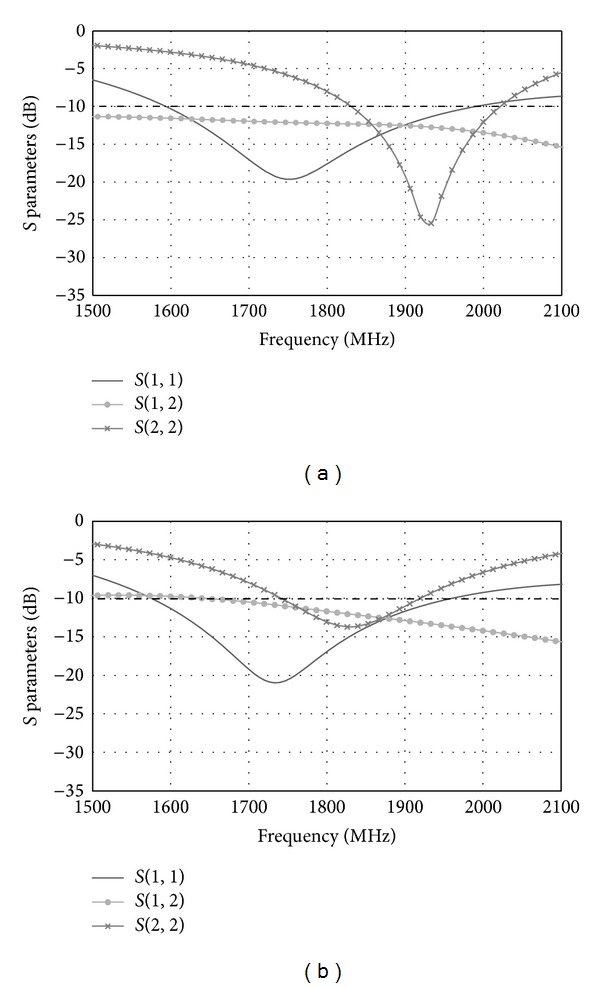
(a) Simulated *S* parameters with *d* = 10.3 mm. (b) Simulated *S* parameters with *d* = 12.3 mm.

Effects of the distance between Port 1 and Port 2 are studied in Figure 13. The simulated *S* parameters graphs for different distances between Port 1 and Port 2 are shown in Figure 13. The results for distance *d* = 10.3 mm, 11.3 mm, and 12.3 mm are simulated. It is found that as the distance *d* increases, the isolation between the two ports decreases. Apart from that, it is observed that changing the value *d* has effects on the resonance frequency of the T-shaped microstrip feedline antenna. As the distance *d* decreases, the resonance frequency of the T-shaped microstrip feedline antenna increases. In order to operate at LTE 1800, the most suitable distance between Port 1 and Port 2 is 11.3 mm.

**Figure 14:**
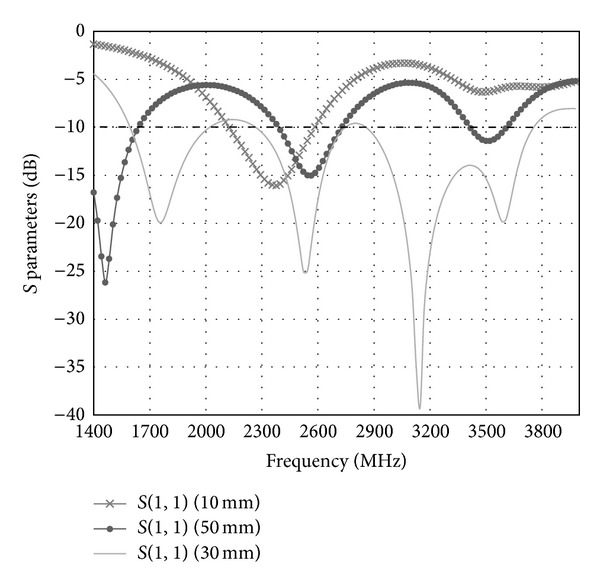
Simulated *S* parameters with the printed monopole's length of 10 mm, 30 mm, and 50 mm.

Figure 14 shows the simulated *S* parameters graph for the printed double-L sleeve monopole antenna only. The *S* parameters (*S*_11_) for the printed monopole's length of 10 mm, 30 mm, and 50 mm are shown in Figure 14. It is found that the length of the printed monopole controls the resonance of the antenna. When the length of the printed monopole is 10 mm, the resonance of the antenna is at 2374 MHz. At the length of 50 mm, two resonance frequencies can be observed at 1464 MHz and 2558 MHz. However, these resonance frequencies cannot operate at LTE 1800. Hence, the length of the printed monopole is chosen to be 30 mm. A large bandwidth of 255 MHz is formed by four resonances obtained from 1680 MHz to 3750 MHz.

**Figure 15:**
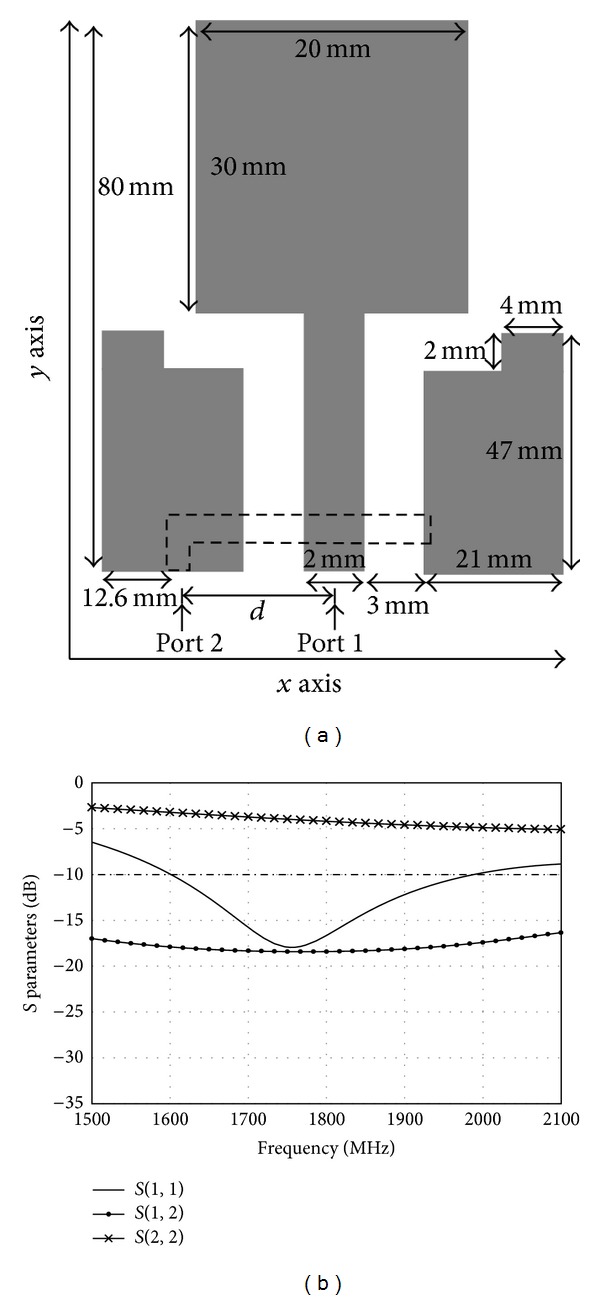
(a) The structure of the T-shape microstrip feedline without the left hand. (b) Simulated *S* parameters with the T-shape microstrip feedline without the left hand.

**Figure 16:**
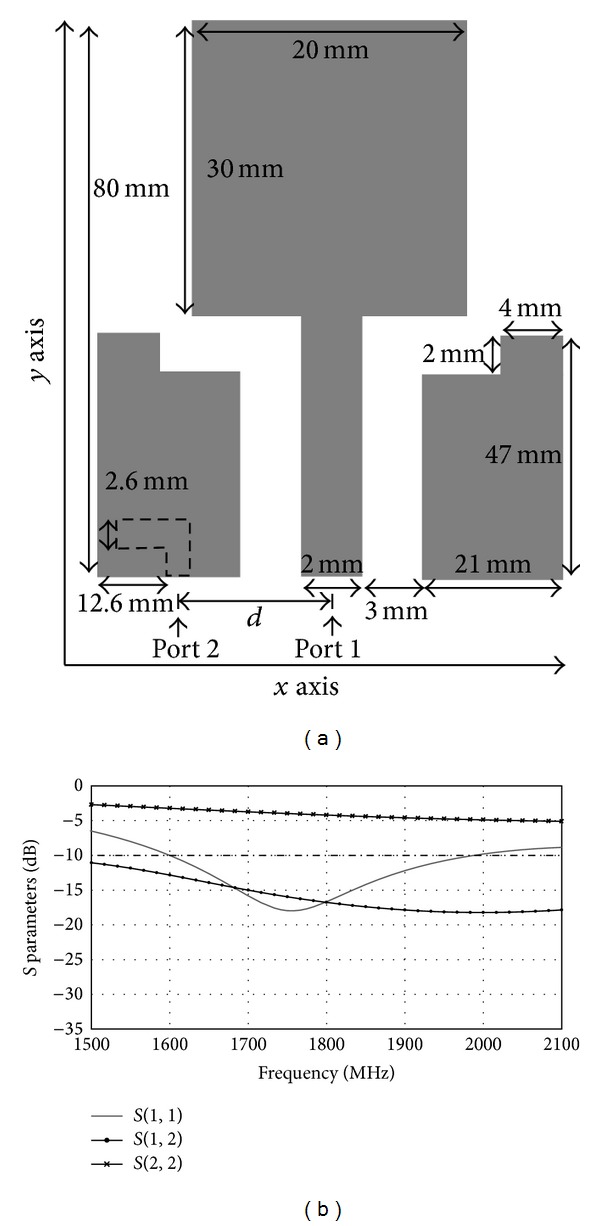
(a) The structure of the T-shape microstrip feedline without the right hand. (b) Simulated *S* parameters with the T-shape microstrip feedline without the right hand.

Figures 15 and 16 show the effects of different shapes of the microstrip feedline. In Figure 15(a), the T-shape microstrip feedline without the left hand is shown.  Figure 15(b) shows that there is no resonance frequency for *S*_22_ in the LTE 1800 range. The structure of the T-shape microstrip feedline without the right hand is shown in Figure 16(a). Similarly, we can see that there is also no resonance frequency for *S*_22_ in the LTE 1800 range in Figure 13(b). The T-shape is crucial to excite the microstrip feedline. The T-shape microstrip feedline antenna has resonance frequency of 1800 MHz with good return loss for *S*_22_ at 21.69 dB as shown in Figure 6(b).

**Figure 17:**
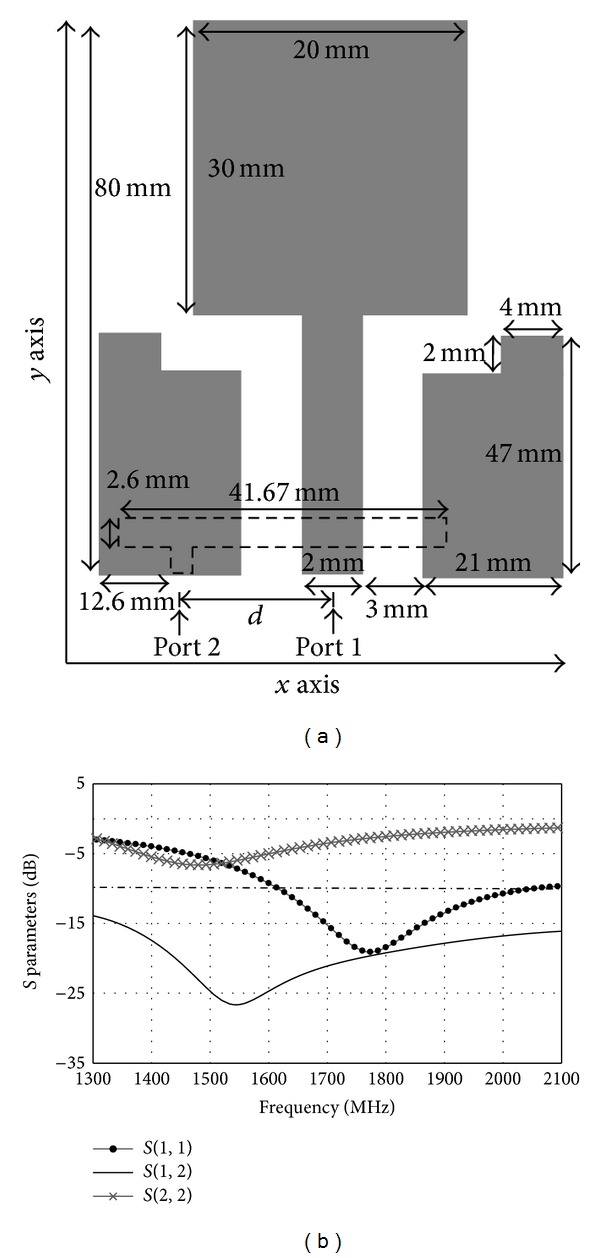
(a) The structure of the antenna with the length of T-shape microstrip feedline being 41.67 mm. (b) Simulated *S* parameters with the length of T-shape microstrip feedline being 41.67 mm.

Figure 17(a) shows the structure of the antenna when the length of T-shape microstrip feedline equals quarter wavelength (41.67 mm). In Figure 17(b), the simulated result for the length of T-shape microstrip feedline that equals quarter wavelength is shown. It can be observed that when the length of the microstrip feedline is equal to quarter wavelength, the resonance frequency is at 1480 MHz and the return loss is 6.64 dB. The length of the T-shape microstrip feedline is fine-tuned so that it can operate at LTE 1800. It is found that when the length of the microstrip feedline is 81.6% of the quarter wavelength (34 mm), the T-shape microstrip feedline antenna can operate at LTE 1800. The simulated results are shown in Figure 6(a).

## 4. Conclusion

A MIMO antenna that can operate in LTE 1800 is presented in this paper. The combination of printed double-L sleeve monopole antenna and T-shaped microstrip monopole feedline antenna contributes to the good isolation characteristics in this proposed antenna. The proposed MIMO antenna also has high gain and efficiency. It is a promising candidate to be integrated in personal digital assistant, tablets, and other wireless electronic devices.
